# Professionals’ roles in the hospital discharge of older adults in 3 Nordic cities: a vignette study

**DOI:** 10.1093/eurpub/ckac130.109

**Published:** 2022-10-25

**Authors:** AEM Liljas, J Pulkki, NK Jensen, B Burström, I Keskimäki, I Andersen, E Jämsen, J Agerholm

**Affiliations:** 1 Global Public Health, Karolinska Institutet, Stockholm, Sweden; 2 Faculty of Social Sciences, Tampere University, Tampere, Finland; 3 Department of Public Health, Copenhagen University, Copenhagen, Denmark

## Abstract

**Background:**

The hospital discharge process of older adults in need of both medical and social care post hospitalisation requires extensive care coordination. Cooperation and continuity between involved care providers are essential, however, existing care systems including the Nordic care systems, are poorly designed to provide health and social care to patients with complex health and social care needs which increases the risk of certain groups not receiving optimal care.

**Aim and methods:**

This study aims to examine and compare what roles, responsibility and actions nurses take in the hospital discharge process of older adults with complex care needs in three Nordic cities: Copenhagen (Denmark), Stockholm (Sweden) and Tampere (Finland). A vignette study consisting of three fictive cases was conducted face-to-face with nurses in Copenhagen (n = 11), Stockholm (n = 16) and Tampere (n = 8). Participants were identified through the researchers’ networks and snowball sampling. The vignettes represent older patients with age-related medical conditions of which one also has cognitive loss and one looks after their partner with dementia. The cases further include differences in the home help received by their children, physical obstacles in their homes and unwillingness of becoming a burden to the system. A thematic approach is used for the data analysis.

**Results and conclusions:**

Preliminary results suggest that the informants’ roles and engagement in the coordination and collaboration may differ both within and between the systems studied, and that they take responsibility beyond their job roles particularly if the patient has no close relatives. The study is of public health importance as it identifies gaps in how the care is organised in the three welfare states targeted. It also sheds light on the complexities of providing universal care in ageing societies where a growing proportion of older adults have both medical and social care needs.

**Key messages:**

- This study reveals similarities and differences in how the hospital discharge process is organised in 3 Nordic cities.
- Gaps in the care system are partly filled by nurses who do work beyond their job roles.

